# Chronic Renal Failure Presenting for the First Time as Pulmonary Mucormycosis with a Fatal Outcome

**DOI:** 10.1155/2015/589537

**Published:** 2015-01-13

**Authors:** B. Jayakrishnan, Jamal Al Aghbari, Dawar Rizavi, Sinnakirouchenan Srinivasan, Ritu Lakhtakia, Dawood Al Riyami

**Affiliations:** ^1^Department of Medicine, Sultan Qaboos University Hospital, 123 Muscat, Oman; ^2^Department of Anaesthesia, Sultan Qaboos University Hospital, 123 Muscat, Oman; ^3^Department of Pathology, College of Medicine and Health Sciences, Sultan Qaboos University, 123 Muscat, Oman

## Abstract

Pulmonary mucormycosis is an uncommon, but important, opportunistic fungal pneumonia which is often diagnosed late. Renal failure as the predominant presenting feature is not common in mucormycosis. Moreover, sudden, massive hemoptysis is not a usual complication. In this report we describe fatal pulmonary mucormycosis in a young patient with a previously undiagnosed chronic renal failure.

## 1. Introduction 

Mucormycosis, an uncommon invasive fungal infection, occurs predominantly in debilitated or immunosuppressed hosts. The conditions predisposing to mucormycosis include malignant hematological disease, prolonged and severe neutropenia, poorly controlled diabetes mellitus with or without diabetic ketoacidosis, iron overload, major trauma, prolonged use of corticosteroids, illicit intravenous drug use, neonatal prematurity and malnourishment, and chronic renal insufficiency [1]. However, mucormycosis has been described in previously healthy individuals as well [2, 3]. Pulmonary mucormycosis has been reported in renal failure, either as a part of chronic uremia or after transplantation [2, 3]. In a literature search spanning 30 years, 13% of the patients with pulmonary mucormycosis had renal disease of which 55% were posttransplant patients [3]. It almost always occurs in patients with an established renal disease. Renal involvement can also occur as a part of a disseminated disease. Mucormycosis has not been reported as the initial presentation of chronic renal failure. The mortality rate is often high, 65% with isolated pulmonary mucormycosis, 96% for those with disseminated disease, and 80% overall. Moreover, mucormycosis is an unusual cause of massive hemoptysis [4, 5]. Here we report fatal pulmonary mucormycosis as the initial presentation of chronic renal failure.

## 2. Case Report

A 26-year-old male expatriate was brought to the emergency of Sultan Qaboos University Hospital, Muscat, Oman, in severe respiratory distress. He was having vomiting, shortness of breath, productive cough, and mild fever for almost a week. There was no history of any previous illness.

**Figure 1:**
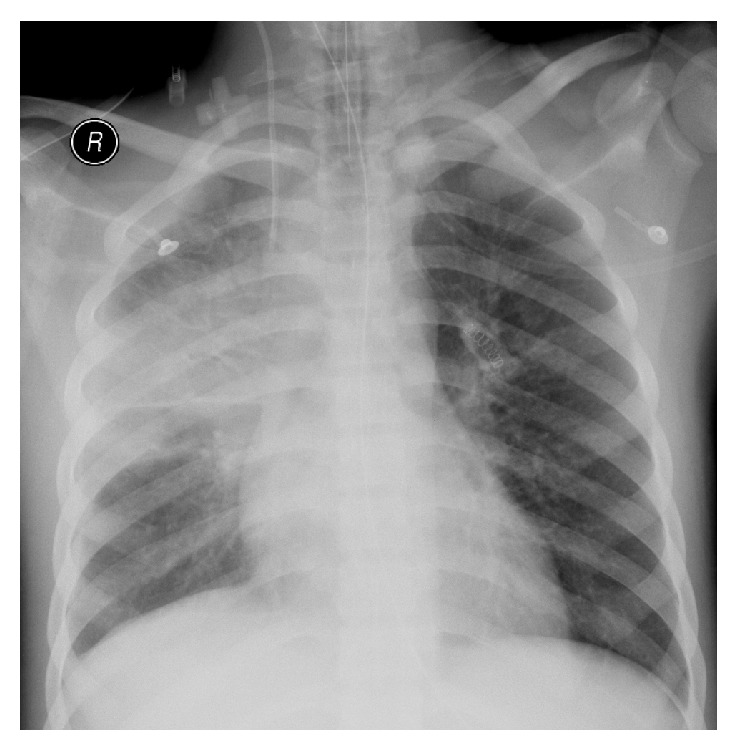
Chest radiograph showing a right mid zone consolidation and a slightly blunt right costophrenic angle.

On arrival he was in severe distress with a respiratory rate of 44/minute, heart rate of 103/minute, and blood pressure of 148/97 mmHg. Arterial blood gas analysis while on oxygen showed severe metabolic acidosis {pH—6.9, PCO_2_—12 mmHg, PO_2_—600 mmHg, and HCO_3_—4.4 mmol/L}. Creatinine and urea were very high and the hemoglobin was very low. The basic blood test results were as follows: fasting blood sugar—4.9 mmol/L; creatinine—1327 *μ*mol/L; urea—46.8 mmol/L; bicarbonate—2 mmol/L; sodium—119 mmol/L; potassium—5.8 mmol/L; glomerular filtration rate—4 mL/min/1.73 m^2^; anion gap—22 mmol/L; calcium—2.18 mmol/L; phosphate—3.98 mmol/L; lactate—0.7 mmol/L; hemoglobin—5.4 g/dL; white cell count—52 × 10^9^/L; lactate dehydrogenase—378 U/L; creatine kinase—2548 U/L; INR—1.29; activated partial thromboplastin time—69.2 seconds. Urine dipstick showed the presence of proteins, glucose, and red blood cells. Chest radiograph showed consolidation in the right mid zone and slight blunting of the right costophrenic angle (Figure 1).

**Figure 2:**
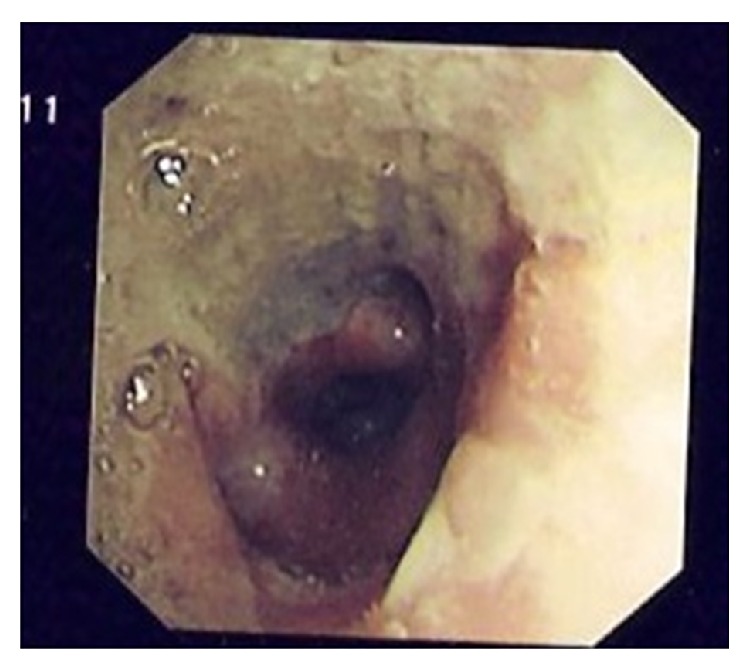
Bronchoscopy showing inflamed right bronchi and the subdivisions lined by a thick layer of yellow secretions.

**Figure 3:**
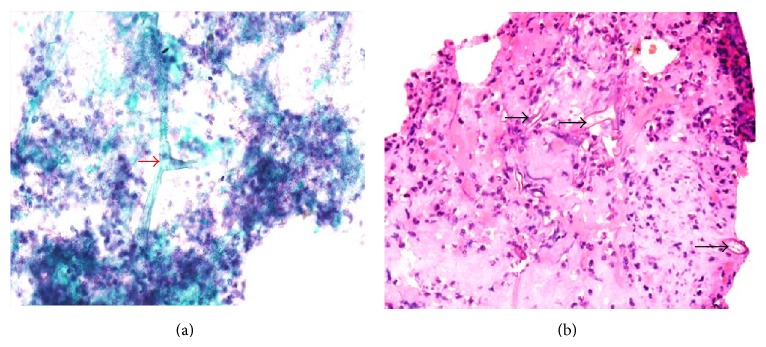
Bronchoalveolar lavage specimen showing broad, irregular, aseptate hyphae of* Mucor* with wide angled branching (arrow-heads) in a neutrophil-rich inflammatory background. (a) Papanicolaou (smear) ×600 and (b) haematoxylin and eosin (cell block) ×600.

The grossly elevated creatinine and urea, low hemoglobin, severe metabolic acidosis, bilateral small kidneys in ultrasound scan of the abdomen, and a high white cell count suggested a primary renal involvement complicated by a pneumonic illness and possibly sepsis. He deteriorated rapidly and was electively intubated and mechanical ventilation was initiated. He received supportive care, fluids, measures to reduce potassium, and broad spectrum antibiotics. Since the acidosis and the renal function did not show any improvement, he was taken up for dialysis later on the same day. Bronchoscopy showed inflamed right bronchi and the subdivisions lined by a thick layer of yellow secretions (Figure 2). The bronchial washings and brushings showed broad aseptate hyphae with right angled branching consistent with* Mucor* species (Figure 3).

Amphotericin (liposomal amphotericin B, 7 mg/kg/day) was added along with the broad spectrum antibiotics which he was receiving since admission. He continued to have dialysis on a regular schedule. Though there was mild improvement in the clinical and metabolic parameters, he continued to be critically ill. He was extubated on the seventh day of admission and was shifted to the ward once the vitals and the level of consciousness were stable. Two days later during dialysis he suddenly developed hypotension. The patient was conscious and communicating and the blood pressure picked up with inotropes. However, he suddenly developed massive hemoptysis. Though he was reintubated and cardiopulmonary resuscitation was initiated he could not be resuscitated.

## 3. Discussion

Mucormycosis is an invasive fungal infection caused by members of the family Mucoraceae and occurs predominantly in debilitated or immunosuppressed hosts. Mucormycosis is an uncommon disease, even in high-risk patients, and represents 8.3%–13% of all fungal infections encountered in such patients [1]. Six predominant clinical forms of the disease exist, which are, in decreasing frequency, rhinocerebral, pulmonary, disseminated, cutaneous, gastrointestinal, and uncommon rare forms [1].

Mucormycosis has been reported in patients of chronic renal failure on treatment as a complication or a terminal event. However, it is rare in an undiagnosed renal failure. Patients with chronic renal failure on maintenance hemodialysis and those receiving deferoxamine therapy for aluminum toxicity have been reported to be more susceptible to mucormycosis. Renal transplant recipients on conventional immunosuppressive therapy are also more prone to develop mucormycosis with an incidence varying from 0.4 to 2% [2, 6]. Mucormycosis usually occurs in the first year after renal transplant [2]. Isolated renal mucormycosis has occurred in intravenous drug users as well as renal transplant recipients in developing countries with warm climates such as India, Egypt, Saudi Arabia, Kuwait, and Singapore [1].

Studies have shown that renal failure portends a poor outcome, and neutropenia in patients with mucormycosis was clearly a predictor of death [3]. Renal involvement has been reported in up to 22% patients with disseminated mucormycosis, but isolated involvement is rare. In a review of 49 patients published in 1971, 10% had uremia. Renal failure is almost universal in patients with bilateral renal involvement [6]. Gupta et al. reported a patient presenting with renal failure and recent GI bleed who had disseminated disease including kidney involvement [7]. In a series of nine cases of fatal disseminated mucormycosis, four patients had chronic renal failure while five had acute renal failure: only two of the latter had proven renal involvement [8]. Primary mucormycosis of the renal allograft is a dreaded disease with a grave prognosis [9, 10]. Interestingly, our patient had features of renal failure and mucormycosis on first presentation.

An immunodeficient state in renal failure seems to be the major factor responsible for increased vulnerability to invasion by opportunistic infections. Decreased cell-mediated immunity and impaired neutrophil function have been documented in renal failure for a long time [11]. In addition, the accompanying acidosis increases the susceptibility to mucormycosis since the iron required for hyphal growth is released from transferrin as the blood pH drops [12]. Specific host immune defects predispose to different forms of mucormycosis. Patients with diabetic ketoacidosis are prone to develop rhinocerebral form and pulmonary mucormycosis typically affects severely immunocompromised individuals.

A hallmark of mucormycosis is extensive angioinvasion with resultant vessel thrombosis and tissue necrosis. Interaction of Mucorales spores with endothelial cells appears to play a critical role in angioinvasion [13]. Sudden, massive hemoptysis is a common fatal complication [4, 5]. The most common causes of death are fungal sepsis (42%), respiratory insufficiency (27%), and hemoptysis (13%).

Our patient had no apparent previous illness. He thus presented for the first time with features of a pulmonary infection and advanced renal failure. This young patient's acute presentation and rapid deterioration are likely due to the pulmonary mucormycosis in the background of chronic renal failure. There was no evidence of renal mucormycosis or disseminated disease from the available evidence. It would be logical to conclude that he had chronic renal failure (undiagnosed/neglected) and the severe fungal infection brought him to the hospital. Tough, sudden, massive hemoptysis is not usual; it is a common cause of mortality in patients with pulmonary mucormycosis.
